# Profiling Suicide Exposure Risk Factors for Psychological Distress: An Empirical Test of the Proposed Continuum of Survivorship Model

**DOI:** 10.3389/fpsyt.2021.692363

**Published:** 2021-07-05

**Authors:** Navjot Bhullar, Rebecca L. Sanford, Myfanwy Maple

**Affiliations:** ^1^School of Psychology, University of New England, Armidale, NSW, Australia; ^2^School of Social Work and Human Service, Thompson Rivers University, Kamloops, BC, Canada; ^3^School of Health, University of New England, Armidale, NSW, Australia

**Keywords:** continuum of survivorship, latent profile analysis, suicide exposure, closeness, impact, time since death, frequency of contact, psychological distress

## Abstract

The Continuum of Survivorship proposes a way in which individuals may experience the suicide death of someone known to them along a continuum from being exposed to the death through to long-term bereavement. The present study provides a first empirical testing of the proposed model in an Australian community sample exposed to suicide. Using a Latent Profile Analysis, we tested the suicide exposure risk factors (time since death, frequency of pre-death contact, reported closeness, and perceived impact) to map to the Continuum of Survivorship model. Results revealed identification of five profiles, with four ranging from suicide exposed to suicide bereaved long-term broadly aligning with the proposed model, with one further profile being identified that represented a discordant profile of low closeness and high impact of suicide exposure. Our findings demonstrate that while the proposed model is useful to better understand the psychological distress related to exposure to suicide, it cannot be used as “shorthand” for identifying those who will be most distressed, nor those who may most likely need additional support following a suicide death. Implications and future research directions are discussed.

## Introduction

With over 3,300 suicide deaths occurring in Australia in 2019 (1) and nearly one million suicide deaths worldwide, suicide is generally recognized as a major public health issue (2). Globally, suicide accounts for 1.4% of all deaths; it the 15th leading cause of death among all age groups and the second leading cause of death among youth (2, 3). Until recently, accurate data examining exposure to suicide among the general population has been lacking (4). Emerging research indicates the prevalence of suicide exposure is far greater than the estimate of six previously offered (5), with up to 135 people affected by each death (6). A recent meta-analysis based on population-based research indicated that past-year exposure to suicide was 4.31% and life-time prevalence of exposure to suicide was 21.83% (7). Additionally, findings from the 2016 General Social Survey in the United States found that 51% of the respondents in this representative sample reported knowing at least one person who died by suicide in their lifetime (8), with ~35% of all respondents were identified as “bereaved,” defined as the respondent indicating that the death was to some extent or very distressing.

Continued methodological (9) and ethical (10) issues in designing prospective studies on impact following exposure to suicide (given it is still a rare and unpredictable event) exist. This is compounded by an ongoing interest in whether bereavement following suicide is similar or different to other forms of unexpected or traumatic death. Over the decades, researchers continue to find that exposure to suicide death is quantitatively similar, yet qualitatively different (11). Nevertheless, deleterious effects from exposure to suicide are numerous and well-reported and include poor mental health outcomes, such as depression and anxiety (12), and suicide risk in both kin (13) and non-kin (4).

To better appreciate who is exposed and affected by suicide death, Cerel et al. (14) proposed a Continuum of Survivorship^1^ theoretical model illustrating the possibility of varying levels of impact to suicide death in the general population. The Continuum model suggests that there is a large number of people who are exposed to every suicide death. Defined as “anyone who knows or identifies with someone who dies by suicide,” the Suicide Exposed group in the Continuum model is hypothesized to be the largest group and may include first responders, community members, acquaintances, colleagues, or fans of celebrities and high-profile public figures, for example (p. 594). Cerel et al. suggest that the effects of suicide exposure for the Suicide Exposed group are likely of low intensity and short duration. Many of those exposed to the suicide will go on to be affected, meaning that their life is at least temporarily disrupted by the death. This category—called Suicide Affected—includes people who experience significant distress but may not be considered bereaved, such as people who witness a suicide or are predisposed to an intense reaction due to pre-existing circumstances (e.g., their own mental health issues).

Further, a smaller number will go on to be Suicide Bereaved (short-term), experiencing a major or devastating life disruption as a result of the death though for a short period of time, or Suicide Bereaved (long-term), meaning that the life disruption continues for a considerable amount of time after the death. It is proposed that those most affected (i.e., bereaved in the short- and/or long-term) will be in close relationship with the person now deceased, including family, extended kin, and friends.

Where such links between a suicide death and the impact on others has been the focus of research, the aim has primarily been bereavement focused, as evidenced in the Continuum model. Rightly, this model demonstrates that suicide impacts many more people than those who are bereaved, yet simultaneously proposes that those impacted most by the exposure to suicide are bereaved. Bereavement, within the traditional understanding of the concept, requires the loss of a significant relationship, typically defined as parents, partners, siblings, children, and friends, and the grief associated with the loss of a loved one is the focus of the bereavement (15). Yet, when examining the breadth of exposure to suicide, many people are exposed, and significantly impacted from that exposure, beyond those grieving a loss. A clear example of this is those who are occupationally exposed to suicide, including health care professionals (16) and first responders such as firefighters (17), ambulance staff (18), and law enforcement officers (19). Additionally, community members who find the deceased when a suicide occurs in public, referred to as zero responders, may not ever have known the person prior to their death, yet still experience impact resulting from the exposure (20). Seemingly, the important suicide exposure risk factors for psychological distress are the self-perceived impact of the death and reported closeness of the relationship. Further, the closer proximity to the death is likely to be a period of heightened distress, as is the frequency of pre-death contact (21, 22).

To date, no empirical research has examined a profile-based approach to map empirical data testing the Continuum of Survivorship model, and how the proposed survivorship profiles are associated with psychological distress. Identification of such typologies of survivorship can provide new insights into how different risk factors combine or co-exist within an individual and how each of these survivorship profiles are related to psychological distress. Traditional variable-centered statistical approaches examine the relationships between variables and results are at the variable-level, thus limiting our ability to form inferences about individuals (23, 24). The point in case is a standard regression variable-based approach that explores the main effects in addition to any interactions, but it does not guarantee that the implied “groups” (with high scores on one variable and low on another) obtained in a regression-based moderation analysis are always meaningful. On the other hand, person-centered approaches, such as a *latent profile analysis* (LPA) groups individuals into homogenous probability-based groupings and examines the relationships between individuals and their different patterns of responses (25).

LPA specifically helps identify specific combinations of variable scores that occur naturally within a sample and classify respondents with similar scores across a set of variables. LPA provides a novel approach to examine the prevalence of different patterns of responses on a range of individual difference variables in a sample (24, 26, 27). Accordingly, we adopted this approach to empirically test the Continuum of Survivorship model by answering the following research question: What survivorship typologies exist and how are these related to psychological distress?

## Materials and Methods

### Participants and Procedure

An online survey was distributed through existing networks by a national peak suicide prevention organization, Suicide Prevention Australia from April through August 2016. Due to this recruitment procedure, we do not know the reach of the survey nor the response rate. Ethics approval was obtained through the University of New England [Approval number HE16-030].

A total of 3,220 unique participants (as per Internet IP address) responded to the survey, 874 cases were excluded for not meeting the inclusion criteria (152 participants reporting no exposure to suicide; 58 cases provided no further information about the nature or impact of the death exposure, 657 cases with extensive missing data, and 7 cases were under 18 years of age). This resulted in a final sample of 2,346 participants who reported exposure to suicide death and provided full data on key study variables included in the LPA analysis. Full details are reported elsewhere (22).

The mean age of participants in the final sample was 44.58 years (age range = 18–86, *SD* = 11.98). Our sample comprised 78.9% of women, 20.2% men; 0.7% other, and 0.2% preferred not to report their gender. It is common across suicide research to have higher female than male respondents (9). Just over half of the respondents (53%) lived in a metropolitan area, 29.5% in regional, 14.2% in rural, 3.2% in remote areas, with 0.2% not reporting their location. Majority of the sample (92.2%) reported not of Aboriginal and Torres Islander (ATSI) descent with 7.6% ATSI participants and 0.2% did not provide data.

### Measures

To examine the variables likely to contribute to an individual being psychologically distressed by exposure to suicide into latent profile groupings, we utilized the following measures from the survey: Time since the person's death, frequency of pre-death contact, closeness to the person, perceived impact of the person's death and psychological distress. Where multiple exposures to suicide attempt and death were reported, participants were asked to answer in relation to the death they regarded as the most impactful to them. Cronbach's α is only reported for measures comprising 2 or more items.

#### Time Since the Person's Death

Participants were asked to report how long since the person died by suicide (in weeks, months, or years). For the analysis, time since death was converted into one single unit as in years.

#### Frequency of Contact

Participants reported the frequency of their contact with the person who died by suicide in the 6 months prior to the death. Contact frequency was assessed on a 6-point scale ranging from 1 = daily to 6 = infrequently. This item was reverse scored so that higher score indicates more frequent contact.

#### Closeness With the Person Who Died

Using 1-item closeness scale (28), participants reported their closeness to the person whose suicide death was most impactful. Closeness was assessed on a 5-point Likert scale ranging from 1 = not close to 5 = very close.

#### Perceived Impact of the Suicide Death

We used 1-item to assess perceived impact for the most impactful death exposure (28). Impact was assessed on a 5-point Likert scale ranging from 1 = had little effect on my life to 5 = had significant/devastating effect on me that I still feel.

#### Psychological Distress

A 10-item measure Kessler-10 (29) was used to assess psychological distress in suicide exposed and bereaved participants. K10 asks participants to identify how often they experienced the problem (i.e., tiredness, nervousness, and hopelessness) in the last 30 days. Items are assessed on 5-point Likert scale ranging from 1 = none of the time to 5 = all of the time) and are summed with higher scores indicating greater levels of distress. Scores on the K10 range from 10 to 50. The Australian Bureau of Statistics (30) categories provide a population level comparison group, being 10–15 = low levels of distress; 16–21 = moderate levels of distress; 22–29 = high levels of distress; and 30–50 = very high levels of distress. Cronbach's α in the present study was 0.94, indicating an excellent internal reliability.

### Statistical Analyses

A LPA using Mplus8.3 (31) was conducted to classify respondents based on shared pattern of their responses on a range of risk factors for suicide. LPA is considered a sophisticated analytical tool used to assess how unique combinations of continuous latent variables and underlying categorical latent variables cluster within homogeneous groupings within a sample. Several model fit indices were assessed to determine the optimal profile model, including the Bayesian Information Criteria (BIC), which assesses improvement in fit after adjusting for the number of parameters in a model, sample size adjusted BIC (32, 33), Vuong-Lo-Mendel-Rubin (VLMR) Adjusted test, and the Bootstrapped Likelihood Ratio test (BLRT). The VLMR and BLRT assess difference in goodness-of-fit between model *k* and model *k*−1, where *k* refers to the number of retained profiles. The preferred model is indicated by a combination of smallest BIC and adjusted BIC values with highest number of profiles, and significant *p*-values for LMR and BLRT indicate best fit, i.e., model *k*−1 should be rejected in favor of model *k* (31). Entropy was also used as an index of model assessment, with values close to one considered ideal (34). In addition to statistical adequacy, we also considered theoretical conformity and meaningfulness and interpretability of the preferred profile-solution to guide our decision regarding retaining the number of profiles (35–37).

To facilitate interpretation of profiles, we standardized the four profiling variables to a mean of 0 with a standard deviation of 1. A multivariate analysis of variance (MANOVA) was conducted to determine significant profile differences in the risk factors (used as profiling variables) and psychological distress (DV). Finally focused chi-squared contingency tests were conducted to examine the proportion distribution of kin/non-kin and gender across the profile membership.

## Results

### Descriptive Statistics

Table 1 shows intercorrelations, means, and standard deviations of key study variables within the LPA sample. More time passed since death was significantly associated with high frequency of contact, and less perceived impact for the most impactful death exposure and psychological distress. However, time since death was not significantly associated with reported closeness to the person who suicide death was most impactful. As expected, high frequency of contact with the person was significantly associated with reported closeness, perceived impact, and psychological distress. Reported closeness with the person was also significantly associated with greater impact and psychological distress, and more impactful the suicide death was, greater the psychological distress.

**Table 1:** Intercorrelations among key study variables.

**Variables**	**1**.	**2**.	**3**.	**4**.	**5**.
1. Time since death	–	0.05^*^	0.01	−0.08^***^	−0.10^***^
2. Contact frequency		–	0.63^***^	0.50^***^	0.14^***^
3. Closeness			–	0.72^***^	0.14^***^
4. Impact				–	0.23^***^
5. Psychological distress					–
Mean	9.10	3.25	3.39	3.67	20.78
*SD*	9.63	1.94	1.46	1.26	8.78

*N = 2346*.

* *p < 0.05*,

*** *p < 0.001*.

On average, participants reported 9 years since the suicide death occurred. The mean scores for frequency of contact (assessed on a 6-point scale) and reported closeness with the person who died (assessed on a 5-point scale) was just below the mid-point. On the other hand, the mean score for perceived impact of the suicide death was just above the mid-point on a 5-point scale. Overall, the study sample reported moderate levels of psychological distress.

### Latent Profile Analysis

To empirically test the Continuum of Survivorship model, we conducted a LPA to identify profiles based on combinations of the four suicide exposure risk factors: time since the person's death, frequency of contact, reported closeness and perceived impact of the person's death. Table 2 provides a summary of various model fit indices for 1- through 7-profile solutions.

**Table 2:** Model fit indices for 1- through 7-profile solutions.

**Profiles**	**BIC**	***Adj* BIC**	**VLMR**	**BLRT**	**Entropy**
1.	26,685.27	26,659.85	–	–	–
2.	23,633.38	23,592.07	<0.001	<0.001	0.87
3.	22,830.64	22,773.45	<0.001	<0.001	0.89
4.	22,259.46	22,186.38	<0.001	<0.001	0.88
**5**.	**21,911.17**	**21,822.21**	**0.004**	**<0.001**	**0.91**
6.	21,752.29	21,647.45	0.006	<0.001	0.90
7.	21,477.81	21,357.08	0.999	1.00	0.90

*N = 2,346. A combination of lowest BIC and adjusted BIC with highest number of profiles and significant p-values for VLMR and BLRT indicate best fit. Entropy values close to 1 indicate best fit. Best fitting profile solution shown in bold*.

**Figure 1:**
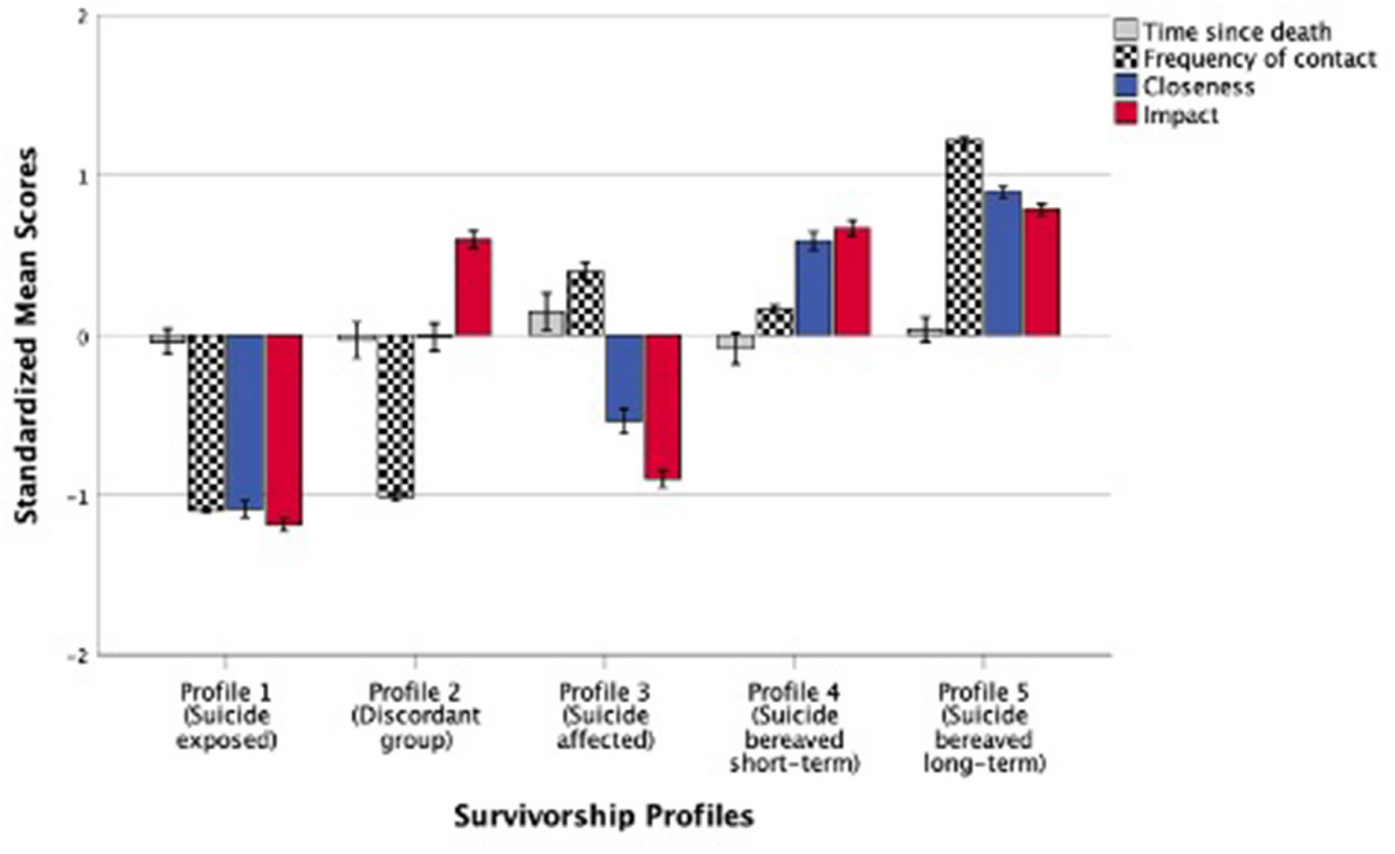
Standardized mean scores (*M* = 0*, SD* = 1) of suicide exposure risk factors across five survivorship profiles. Error bars represent standard errors (SE) ±1.

Results revealed that the 5-profile solution met the criteria for all the relevant fit indices. In addition to the statistical adequacy, our preferred profile solution also demonstrated practical meaningfulness of the profiles mapping onto the Continuum of Survivorship model. Therefore, we interpreted the 5-profile solution in the present study. Figure 1 shows the standardized mean scores of the profiling variables (time since the person's death, frequency of contact, reported closeness and perceived impact of the person's death). Profile 1 (*n* = 603, 25.7% of the sample), labeled as “Suicide exposed” comprised individuals who reported being suicide exposed but reported no impact. Profile 2 (*n* = 352, 15%), labeled as “Discordant group,” comprised individuals who reported discordant patterns of low levels of reported closeness with person but very high levels of perceptions of impact of the suicide death. Respondents in Profile 3 (*n* = 318, 13.6%), labeled as “Suicide affected,” reported above average time since death and frequent contact with the person but low levels of closeness and impact of death. Profile 4 (*n* = 380, 16.2%), labeled as “Suicide bereaved short-term,” comprised individuals reporting frequent contact and high levels of closeness and impact but time since death was more recent. Finally, Profile 5 (*n* = 693, 29.5%), labeled as “Suicide bereaved long-term,” was the largest group in the study comprising individuals who reported the most frequent contact with the person, closeness and severe impact of the person's death and with average time since death.

#### Examining Profile Differences in the Risk Factors and Psychological Distress

We conducted one-way MANOVA to examine profile differences in the suicide exposure risk factors (profiling variables) and psychological distress (DV). Results found a significant profile differences in the four risk factors, *F*_(20, 9, 360)_ = 331.32, *p* < 0.001; Pillai's Trace = 1.66; partial η^2^ = 0.42, a large effect size. *Post-hoc* comparisons, summarized in Table 3, revealed that individuals in Profile 2 reported significantly less time since death than that of Profile 3 who reported the most time elapsed since their person's death. There were no other significant profile differences on this risk factor. In contrast, there were significant profile differences in frequency of contact and reported closeness with the person. Specifically, Profile 1 reported least contact, followed by Profiles 2, 4, 3, and 5, respectively. In terms of reported closeness to the person whose suicide death was most impactful, Profile 1 reported the least closeness followed by increasing closeness as indicated by Profiles 3, 2, 4, and 5, respectively. Respondents in Profile 1 also reported the least impact of the person's death and was significantly different from other profiles, with Profile 5 reporting greatest impact. However, there was no significant difference between Profiles 2 (Discordant group) and 4 (Suicide bereaved short-term). Results based on adjusted standardized residuals from the contingency table analyses suggested significantly greater number of kin relationships in “Suicide bereaved long-term” profile (Profile 5), and significantly greater number of non-kin relationships in “Suicide exposed” profile (Profile 1). There were significantly more females in the “Suicide bereaved-long term” profiling group (Profile 5) and more males in the “Suicide exposed” profile (Profile 1).

**Table 3:** Means, standard errors (SE), and mean differences or distributions across five survivorship profiles.

	**Profile 1 Suicide exposed**	**Profile 2 Discordant group**	**Profile 3 Suicide affected**	**Profile 4 Suicide bereaved short-term**	**Profile 5 Suicide bereaved long-term**	**Univariate**	
	**(*n* = 603)**	**(*n* = 352)**	**(*n* = 318)**	**(*n* = 380)**	**(*n* = 693)**		
**Profiling variables**			**M(SE)**			*F*_(4, 2, 341)_	Partial η^2^
Time since death	8.69^abc^ (0.39)	8.78^abc^ (0.51)	10.50^b^ (0.54)	8.27^c^ (0.49)	9.43^abc^ (0.37)	2.97^*^	0.01
Contact	1.11^a^ (0.02)	1.28^b^ (0.03)	4.01^c^ (0.03)	3.55^d^ (0.03)	5.61^e^ (0.02)	7,135.98^***^	0.92
Closeness	1.79^a^ (0.04)	3.36^b^ (0.05)	2.59^c^ (0.05)	4.24^d^ (0.05)	4.69^e^ (0.03)	1,026.04^***^	0.64
Impact	2.17^a^ (0.02)	4.42^b^ (0.03)	2.53^c^ (0.03)	4.51^b^ (0.03)	4.66^d^ (0.02)	2,113.53^***^	0.78
**Outcome variable**							
Psychological distress	18.68^a^ (0.35)	21.50^bce^ (0.46)	19.47^ac^ (0.48)	20.73^c^ (0.44)	22.87^e^ (0.33)	21.50^***^	0.04
ABS (2012) categories	Moderate	Moderate-high	Moderate	Moderate	High		
**Demographics**							
**Relationship with the person^+^**	***n***						
Non-kin	474	160	282	148	193	$\chi_{\left(4\right)}^{2}$ = 539.38^***^	
Z_Residual_	14.4	−3.2	13.5	−6.2	−16.3		
Kin	126	189	35	230	499		
Z_Residual_	−14.4	3.2	−13.5	6.2	16.3		
**Gender^++^**							
Female	457	290	232	292	579	$\chi_{\left(4\right)}^{2}$ = 20.27^***^	
Z_Residual_	−2.1	1.6	−2.6	−0.9	3.4		
Male	139	60	81	83	110		
Z_Residual_	2.1	−1.6	2.6	0.9	−3.4		

*Means in rows with different superscripts are significantly different using Bonferroni adjustment for multiple comparisons. Time since the person's death measured in years. Frequency of contact assessed on a 6-point scale whereas closeness and impact of the person's death assessed on a 5-point scale*.

* *p < 0.05*,

*** *p < 0.001*.

+ *Data available from 2,336 participants*.

++ *Data available from 2,323 participants (Other = 13, Prefer not to say = 5)*.

*Z_Residual_ = Adjusted standardized residual, where Z_Residual_ = 2 is significant at p < 0.05*.

Finally, profile membership was significantly associated with psychological distress (DV). In particular, Profile 1 reported significantly lower levels of psychological distress than that of Profiles 2, 4, and 5, respectively. However, there was no significant difference in distress levels between Profiles 1 (Suicide exposed) and 3 (Suicide affected). Profile 5 reported significantly highest levels of distress compared with Profiles 1, 3, and 4, respectively. However, there were no statistically significant differences in distress levels between Profiles 2 (Discordant group) and 5 (Suicide bereaved long-term), and between Profiles 3 (Suicide affected) and 4 (Suicide bereaved short-term), respectively. Table 4 provides a summary of matching between the Continuum of Survivorship model and profile typologies identified in the present study.

**Table 4:** Matching the Continuum of Survivorship with profile typologies.

**Continuum model**	**Profile typologies**	**Similarities**	**Differences**
**Suicide exposed**. *Neither close to the person nor experience distress*	**Profile 1: Suicide exposed**. *Report exposure to suicide but no impact nor closeness and least psychological distress. 25.7% of the sample*.	Close alignment between continuum and profile typology	–
**Suicide affected**. *Experience distress, but not grieving loss of attachment*	**Profile 3: Suicide affected**. *Closer to time of death and higher pre-death contact; low closeness and impact. 13.6% of the sample*.	Experience moderate levels of distress	No statistically significant difference in psychological distress compared with “Suicide exposed” profile rather the proximity to the death and contact with the person pre-death.
**Suicide bereaved short-term**. *Grieving the loss of an attached person, including intimate relationships, but do not progress to long term bereavement*	**Profile 4: Suicide bereaved short-term**. *Frequent pre-death contact, high closeness and impact of death, death more recent, increased psychological distress. 16.2% of the sample*.	Experience statistically significant greater levels of distress than “Suicide affected” profile	Unknown at time of data collection whether the greater levels of distress in close proximity to the death will result in progressing to long-term bereavement or not.
**Suicide bereaved long-term**. *Those who struggle for protracted periods of time, aligned with prior “suicide survivors” definitions*	**Profile 5: Suicide bereaved long-term**. *Most frequent pre-death contact, highest closeness and severe impact. Average time since death. Highest levels of psychological distress. 29.5% of the sample*.	Experience statistically significant highest levels distress than any other profile	This is the largest profile group due to sampling procedure. Time since death was not statistically different from other profiles, and was an average time (9 years) rather than the proposed continuum “protracted” time.
No continuum category	**Profile 2: Discordant**. *Low/High group—low closeness and high impact. 15% of the sample*.	–	Discordant group where low closeness but high impact was reported. Less time since death, moderate levels of distress similar to Profiles 3, 4, and 5.

## Discussion

The current study aimed to empirically test the proposed Continuum of Survivorship model (14). This was achieved by conducting a Latent Profile Analysis as a means of establishing combinations of suicide exposure risk factors in a community sample of suicide exposed and bereaved people to determine how these survivorship profiles might be related to psychological distress and whether this fits within the groups proposed in the Continuum model. The present study presents a novel approach within the suicide bereavement literature and highlights the utility of looking beyond variable-level analysis. The main findings and implications are discussed below.

### Profile Segmentation

LPA revealed five distinct survivorship profile typologies: Profile 1 comprised suicide exposed individuals, Profile 2 comprised a discordant group of respondents who reported less closeness but high impact related to the person's death. Profile 3 included suicide affected people, who were experiencing high psychological distress, but for whom the death was more recent. Finally, Profiles 4 and 5 comprised suicide bereaved individuals. When comparing these profiles with the Continuum of Survivorship model there is both confirmation of the model, and deviation from it. The following discussion follows the progression through the model as proposed by Cerel et al. (14) and presented in Table 4. Profile 1 overlaps with the definition of “Suicide exposed” —and was numerically the second largest profile grouping as expected by the Continuum model. These individuals were neither close to the person who died, nor did they report heightened psychological distress. Profile 3 relates to the Continuum group of “Suicide affected,” whereby these individuals report distress in the absence of a close relationship. Conversely, Profile 3 includes higher pre-death contact suggesting a relationship with the person was present, but there was no statistically significant difference in psychological distress to those in Profile 1. Profile 4 is similar to those in the Continuum who are “Suicide bereaved short-term” as the death was more recent. Those in this profile grouping may or may not go on to experience long-term bereavement, yet for whom this occurs is not able to be predicted by the Continuum nor by the current empirical testing using LPA. Suicide bereaved long-term matches our Profile 5 group, however, length of time from death was not statistically significant from the other Continuum groups suggesting that time may not be the important feature for those who are most affected by suicide death exposure. Finally, our results identified a discordant group who are not proposed as a distinct group in the Continuum. This discordant profile grouping identifies those for whom there was high impact from the suicide death exposure with low reported closeness, less time since death and moderate distress levels similar to Profiles 3, 4, and 5. This group requires further investigation to better understand those for whom there is high impact of a death while seemingly not as a result of the loss of an attachment given the low closeness of the relationship.

The present study is the first empirical testing of the theorized Continuum of Survivorship model. Previous research has applied the model to samples of suicide exposed groups by simply overlaying impact of the death to the categories which are relationship based along the continuum. For example, Cerel et al. (14) suggest that an impact scale, such as the one used in this study, could potentially be used as “short-hand to identify people in each of the proposed categories” (p. 598). The results of this study suggest this does not accurately reflect the experiences of all individuals exposed to, and impacted by, suicide. People occupationally exposed to suicide, particularly first responders, are an important example for consideration. Emerging research on the personal and professional impact of exposure to suicide among ambulance personnel suggests that staff experience considerable impact due to the complex challenges associated with experiencing multiple suicide exposures and compassionately responding to people on scene without adequate training to do so, though this distress may be not acknowledged or supressed due to lack of appropriate work-based supports, reluctance to access available support related to concern about confidentiality and competence of support staff, and a culture of stigma associated with asking for help (18). While the Continuum model proposes first responders as individuals who may fit in the categories of suicide exposed or suicide affected given their lack of closeness to the person who died, our results suggest another conceptualization of this experience, where closeness to the decedent is minimal yet the impact of the exposure is significant and life disrupting. We note that our findings align with emerging qualitative research with these groups (16), where the absence of closeness with simultaneous impact suggests that an attachment theory-based model does not adequately explain all responses to exposure to suicide death.

The Continuum of Survivorship model is based on attachment theory, and presumed closeness is evident in the types of individuals proposed to be within each category on the Continuum (14). However, without fully appreciating the complexity of impact and relational closeness within kinship relationships and beyond appears to conflate perceived impact with bereavement based on an assumption of attachment as most significant for the experience of impact. The highest levels of impact are presumed to be indicative of bereavement, though closeness or attachment to the person who died has never before been a consideration in the mapping process. For example, in the General Social Survey (GSS) Feigelman and colleagues (8) included questions to assess exposure to suicide among a representative sample of American adults. The GSS also included a measure of bereavement: “Was that person's death emotionally distressing to you?” For the purpose of analysis, respondents who answered 1) Yes, greatly or 2) Yes, to some extent were coded as “bereaved by suicide,” resulting in 35% of respondents deemed bereaved by suicide due to the reported emotional distress caused by the death. However, a distressing death and resultant bereavement are not the same and our results raise important questions about the presumed equivalence of bereavement and emotional distress, as in the Feigelman et al. (8) study, or impact as in the Continuum model (14). While there are some individuals for whom impact does equate to bereavement, further work is required to unpack the conflation of impact and bereavement commonly reflected in the suicide exposure literature.

Individuals within the discordant profile grouping are not bereaved in the traditional sense of the term, as they are not grieving the death of a close relation, yet they report high levels of impact reflective of significant disruptions to life for either a short- or long-period of time. Many of these individuals may require support to mitigate against potential harms resulting from exposure, yet it is likely that traditional postvention services, such as support groups and bereavement counseling, would not be appropriate given their focus on those bereaved are often family and close friends. For example, individuals in the discordant group with workplace exposure to suicide may experience both personal and professional impacts, such as Vicarious Trauma and Post-traumatic Stress Disorder (38). Trauma-specific interventions, such as Eye Movement Desensitization and Reprocessing (EMDR), have been suggested to mitigate effects of workplace exposure to trauma (39), and may be a more suitable alternative to traditional bereavement counseling interventions which focus on grieving the loss of an attached person. Research is needed to better appreciate the heterogeneous nature of impact and resulting needs among individuals in the discordant group.

The results of the LPA contribute significantly to the ongoing theoretical evolution to assist in understanding why some people are more vulnerable to psychological distress following exposure to suicide regardless of the relationship to the deceased. Our analysis adds empirical testing to the proposed Continuum of Survivorship model. The proposed model usefully explains that for those who lose an attached relationship through suicide are most likely to experience significant distress. However, time since death may not be a useful indicator for those who will continue to experience this significant loss. Utilizing this novel, person-centric analytic approach has also uncovered nuance within those self-identifying as exposed to suicide indicating that there are more complex relationships and impact from exposure to suicide than the continuum currently explains. Importantly, these are individuals who are deserving of our attention, given they are less likely to be in contact with services as they are not the “traditionally bereaved,” and may be reluctant to utilize available resources (20, 40). As we expand our understanding of the discordant group, targeted resources and outreach to individuals based on the nature of impact and their needs may result in greater service utilization.

### Limitations and Future Research Directions

The present study is novel and not without its limitations, therefore, findings should be interpreted with caution. First, the use of a cross-sectional survey design limits any causal inferences about the obtained effects, and the present study used self-report measures, which are susceptible to social desirability. We also acknowledge that the profiles identified in the present study might not reflect existing subgroupings within the actual population (35). The current study design is focused on a point in time profile groupings, future research could employ longitudinal extensions of LPA to track trajectories of survivorship profile membership over time to develop targeted postvention (20). Second, our study comprised a self-selected predominantly white, Australian community sample responding to a request to participate in a survey about exposure to suicide death. Therefore, this may have been more appealing to those who may represent the profile membership in different ways to those for whom exposure to suicide was not a significant event in their life, nor those who are highly distressed. Future research might also replicate the present findings with different age and population groups (including those from First Nations and culturally and linguistically diverse backgrounds), especially the use of a clinical sample is recommended. Third, the ways in which individuals experience multiple suicide exposures remains an area for future investigation as the present study survey asked participants only to focus on the death that was most distressing to them. For whom and in what circumstances result in the discordant profile is a priority for future research. Fourth, the K10 is a measure of global distress and not specific to suicide exposure, and thus future research should examine more nuanced tools, especially assessing suicide exposure distress. Finally, our LPA results identified a discordant profile that did not match with the Continuum of Survivorship model. Further exploration of the experiences of individuals in the discordant profile grouping is necessary to explicate the dimensions of this category and propose revisions to the Continuum model accordingly. In particular, future research could explore the meaning associated with the exposure to suicide and its impact, as others have found the meaning made following exposure to suicide impactful to future vulnerability to suicide (41, 42).

## Conclusion

Overall, our findings contribute a novel approach to the suicidology literature, specifically in relation to better understanding how survivorship profiles correlate with psychological distress. Our study provided the first empirical testing of the Continuum of Survivorship model. We support the use of a profile approach in this area of research, and encourage further research which operationalizes this perspective to move beyond a variable-level approach, so as to capture the multi-dimensionality of an individual's trait combinations and its impact on behavior. Further, terminology that better captures the breadth of experience following exposure to suicide is required beyond survivorship based on assumptions of loss of attachment.

## Data Availability Statement

The raw data supporting the conclusions of this article will be made available by the authors, without undue reservation.

## Ethics Statement

The studies involving human participants were reviewed and approved by Human Research Ethics Committee, University of New England. The patients/participants provided their written informed consent to participate in this study.

## Author Contributions

MM: original project design. NB and MM: conceptualization and methodology. NB, MM, and RS: analysis and final draft. All authors contributed to the article and approved the submitted version.

## Conflict of Interest

MM was a member elected Director on the Board of Suicide Prevention Australia at the time this study survey was disseminated. The remaining authors declare that the research was conducted in the absence of any commercial or financial relationships that could be construed as a potential conflict of interest.

## Footnotes

^1^Survivors of suicide is commonly used in North America to refer to those who have been bereaved by suicide.
